# Optimal sampling interval for characterisation of the circadian rhythm of body temperature in homeothermic animals using periodogram and cosinor analysis

**DOI:** 10.1002/ece3.11243

**Published:** 2024-04-09

**Authors:** Grace Goh, Kristine Vesterdorf, Andrea Fuller, Dominique Blache, Shane K. Maloney

**Affiliations:** ^1^ School of Human Sciences The University of Western Australia Crawley Western Australia Australia; ^2^ Brain Function Research Group, School of Physiology, Faculty of Health Sciences University of the Witwatersrand Johannesburg South Africa; ^3^ School of Agriculture and Environment The University of Western Australia Crawley Western Australia Australia

**Keywords:** chronobiology, core temperature, field monitoring, sampling frequency, thermometry

## Abstract

Core body temperature (*T*_c_) is a critical aspect of homeostasis in birds and mammals and is increasingly used as a biomarker of the fitness of an animal to its environment. Periodogram and cosinor analysis can be used to estimate the characteristics of the circadian rhythm of *T*_c_ from data obtained on loggers that have limited memory capacity and battery life. The sampling interval can be manipulated to maximise the recording period, but the impact of sampling interval on the output of periodogram or cosinor analysis is unknown. Some basic guidelines are available from signal analysis theory, but those guidelines have never been tested on *T*_c_ data. We obtained data at 1‐, 5‐ or 10‐min intervals from nine avian or mammalian species, and re‐sampled those data to simulate logging at up to 240‐min intervals. The period of the rhythm was first analysed using the Lomb–Scargle periodogram, and the mesor, amplitude, acrophase and adjusted coefficient of determination (*R* ^2^) from the original and the re‐sampled data were obtained using cosinor analysis. Sampling intervals longer than 60 min did not affect the average mesor, amplitude, acrophase or adjusted *R* ^2^, but did impact the estimation of the period of the rhythm. In most species, the period was not detectable when intervals longer than 120 min were used. In all individual profiles, a 30‐min sampling interval modified the values of the mesor and amplitude by less than 0.1°C, and the adjusted *R* ^2^ by less than 0.1. At a 30‐min interval, the acrophase was accurate to within 15 min for all species except mice. The adjusted *R* ^2^ increased as sampling frequency decreased. In most cases, a 30‐min sampling interval provides a reliable estimate of the circadian *T*_c_ rhythm using periodogram and cosinor analysis. Our findings will help biologists to select sampling intervals to fit their research goals.

## Graphical abstract

The circadian rhythm of core body temperature is an indicator of the physiological state of a homeothermic animal and can be used as a biomarker of its fitness to its environment. In this study, we investigate the impact of sampling intervals used to collect body temperature data on cosinor estimates of the circadian rhythm, and discuss the optimal intervals required for an accurate estimation of the body temperature rhythm when using data loggers with limited battery and memory capacity in the field.

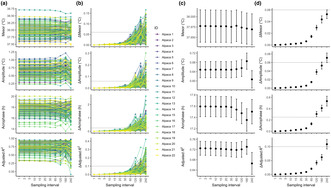

## 1 INTRODUCTION

Core body temperature (*T*_c_) in homeotherms is regulated by a range of autonomic processes, including the production and dissipation of metabolic heat (Jessen, 2001). The precise measurement of *T*_c_ is fundamental to animal research because *T*_c_ exerts a global effect on physiology via its impact on the rate of biochemical reactions and the maintenance of *T*_c_ is a vital function of the autonomic nervous system (Meyer et al., 2017). Although homeotherms actively regulate *T*_c_, most species exhibit a characteristic daily rhythm, typically with a period of higher *T*_c_ during the active phase, and a period of lower *T*_c_ during the inactive, or resting, phase. Given that the daily rhythms can be viewed as smooth waveforms with added noise, a combination of periodogram and cosinor‐based rhythmometry has been used extensively to characterise the circadian rhythm of *T*_c_ by describing the parameters that define a sinusoidal waveform. Specifically, these parameters include the period length, defined as the time taken to complete one cycle; the mesor, which is a measure of the central tendency of the rhythm; the amplitude, which is the difference between the mesor and the maximum or minimum; the acrophase, which describes the time at which the peak of the cycle occurs; and adjusted coefficient of determination (*R* ^2^), that is, the strength of the fit of the sinusoid to the rhythm adjusted for sample size (Bagheri et al., 2007; Refinetti, 2016).

Analysis of the *T*_c_ rhythm offers a valuable and relatively non‐invasive method to assess changes in the circadian rhythm in response to changes in environmental conditions. Changes in the characteristics of the circadian *T*_c_ rhythm can be informative of response and adaptation to physiological, environmental and ecological change. For example, changes in acrophase are observed during arctic‐bound and longitudinal avian migration (Eichhorn et al., 2021). Mesor and amplitude can also be viewed as indices of physiological strain. The *T*_c_ amplitude is inversely correlated with energy intake (Goh et al., 2016; Maloney et al., 2013) and water intake (McCarron et al., 2001; Samara et al., 2012). The mesor and amplitude of *T*_c_ decrease during pregnancy (Crew et al., 2018; Maloney et al., 2017; Trethowan et al., 2016) and are indicative of ovulation (Cagnacci et al., 1996; Crew et al., 2018; Lee, 2016) and the level of sex steroid hormones (Marrone et al., 1976). The maximum daily body temperature has also been used to phenotype the heat tolerance of production animals (Dikmen et al., 2012).

The method of data acquisition is a critical consideration in the study of *T*_c_ and *T*_c_ rhythms. An overview of the instruments typically used to measure *T*_c_ in field and laboratory studies is given in Meyer et al. (2017) and Maloney et al. (2019). Briefly, besides manual thermometers, temperature‐sensitive data loggers, transmitters or transponders can be used to measure *T*_c_. Estimation of the circadian rhythm of *T*_c_ requires repeated sampling over a 24‐h period, and implantable temperature‐sensitive devices are favoured because they facilitate *T*_c_ measurement without the need to repeatedly handle an animal, which can result in stress‐induced hyperthermia (Dallmann et al., 2006).

**FIGURE 1:**
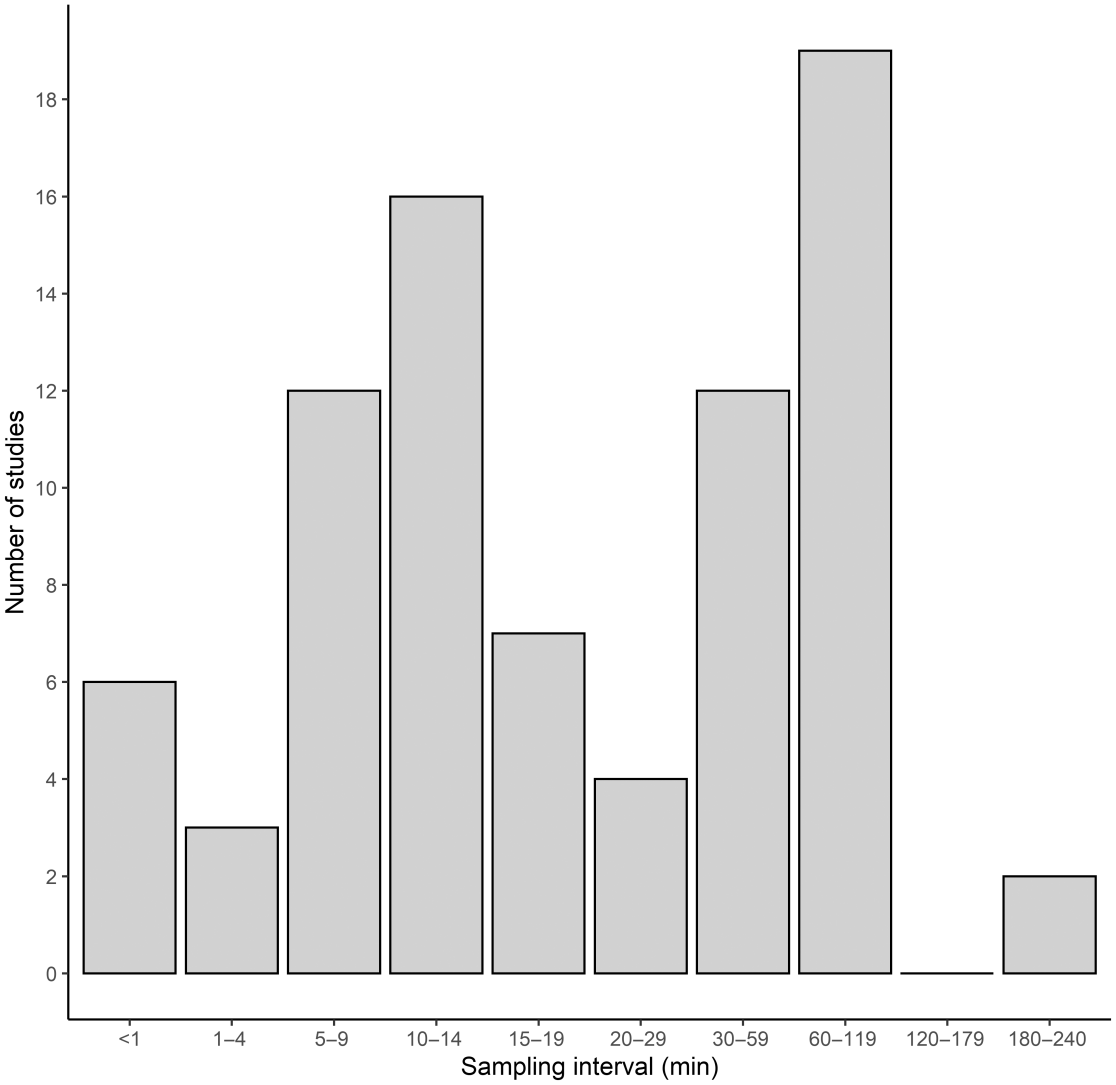
Histogram showing the sampling frequencies used in published field studies that measured *T*_c_ in mammals (*n* = 72). The list of references used to generate the graph is given in the Appendix 2.

After data are collected, they are processed in some way to provide a summary, from calculating a simple daily mean and range (maximum minus minimum) to the fitting of complex models. A body of the literature covers the principles of signal processing theory, which is fundamental to electrophysiology (Nilsson et al., 1993). A particularly relevant concept is the Shannon–Nyquist sampling theorem, which states that a signal must be sampled at more than double the highest frequency that is present in the signal to resolve all frequencies in its function (Shannon, 1949). If that theorem holds for the analysis of the circadian rhythm of body temperature, then three samples per day should suffice to resolve the period. Further, to adequately resolve both the frequency and the amplitude content of a waveform, a general rule of thumb is to sample a given analogue waveform at least three to five times per cycle (Glaser & Ruchkin, 1976). Whether those general rules apply to a signal such as the circadian rhythm of *T*_c_ has never been tested. Logically, continuous *T*_c_ sampling would lead to the greatest signal resolution. Transponder and transmitter‐based telemetry facilitate the near‐continuous measurement of *T*_c_, and developments in technology have greatly improved the transmission range and somewhat mitigated the need for the subject to remain close to its receiver (Lewis Baida et al., 2021). However, the infrastructure required for advanced transmission is costly and difficult to implement in challenging environments. At present, data loggers, which store data that are later retrieved and downloaded, are used most frequently to measure *T*_c_ in unrestrained animals (Maloney et al., 2019). The decision on sampling interval is left to the researcher, who has to counterbalance the finite memory capacity and limited battery life of a logger with the temporal resolution of *T*_c_ that is required for their research. As an example, Figure 1 shows a non‐exhaustive illustration of the sampling intervals used in published studies (*n* = 72) that measured core *T*_c_ of animals in the field using either data loggers (*n* = 64) or radio transmitters (*n* = 15). Of these, two groups that used data loggers stated explicitly that their logging interval was chosen because of a desired logging period (Bieber et al., 2014; Hoelzl et al., 2015) or to maximise the total logging period (Nicol & Andersen, 2008). Cosinor analysis is robust against non‐equidistant sampling and biases caused by acute changes in *T*_c_ (Cornelissen, 2014), but researchers need guidance to know the minimum sampling interval that is required to obtain robust data. It has been suggested that sampling more often than the Nyquist rate does not improve electromyography signals (Ives & Wigglesworth, 2003), but the degree to which the sampling interval affects the output of periodogram and cosinor analysis of *T*_c_ remains unknown.

The aim of this study was to establish the optimal sampling interval that is required to obtain an accurate estimate of the period, mesor, amplitude, acrophase and adjusted *R* ^2^ values that describe the circadian rhythm of *T*_c_. Body temperature data obtained from nine homeothermic species spanning a body mass range from 30 g to nearly 200 kg, and including birds and mammals, were re‐sampled to simulate different sampling intervals, and the periodogram and cosinor variables derived from the re‐sampled data were compared to those derived at the original sampling interval to determine the interval at which the periodogram or cosinor model diverged from the original estimate. Based on the results, recommendations of appropriate sampling intervals for the purpose of periodogram and cosinor analysis are made.

## 2 METHODS

Five continuous days of core *T*_c_ data collected from previous studies on alpaca, cheetah, mouse, barnacle goose, Pekin duck, rabbit, rat, sheep and blue wildebeest were used for analysis. The 5 days of data were selected when the animals were not challenged by known environmental factors or by experimentation. The *T*_c_ data were re‐sampled to simulate data collection at different sampling intervals. For the original data collected at 1‐min intervals, the *T*_c_ data were re‐sampled at 2‐, 5‐, 10‐, 15‐, 20‐, 30‐, 60‐, 120‐, 180‐, and 240‐min intervals (alpaca, cheetah and mouse). The data collected at 5‐min intervals were re‐sampled at 10‐, 15‐, 20‐, 30‐, 60‐, 120‐, 180‐, and 240‐min intervals (barnacle goose, Pekin duck, rabbit, rat and sheep), and the data collected at 10‐min intervals were re‐sampled at 20‐, 30‐, 60‐, 120‐, 180‐ and 240‐min intervals (blue wildebeest). Further information about the experiments can be found in the cited references where available.

### 2.1 Body temperature data

#### 2.1.1 Alpaca

Twenty‐two adult male alpacas (*Vicugna pacos*, mean body mass ± SD: 47.4 ± 8.7 kg) were kept in outdoor paddocks at Banksia Park Alpaca Stud, Serpentine, Australia. Each alpaca had a miniature temperature logger (mlog_T1A, resolution 0.06°C, calibrated accuracy <0.1°C, Sigma Delta Technologies, Australia) implanted into the abdominal cavity that measured *T*_c_ every minute. The temperature loggers had a storage capacity of 2 Mb and a measurement range of 0–60°C. The temperature loggers were waterproofed with biologically inert wax (Sasol EXP986, Sasol Chemical Industries Ltd., Johannesburg, South Africa) and calibrated pre‐ and post‐experiment in an insulated water bath (Haake DC30, Germany) against a certified thermometer (Quat 100, Hereaus, Germany, certification by the National Association of Testing Authorities, Australia) to an accuracy of better than 0.05°C. The loggers were soaked in chlorhexidine solution for at least 24 h prior to surgery. Surgery for logger implantation was performed with the alpacas under deep anaesthesia. The temperature loggers were implanted into the peritoneum via a 5‐cm incision in the right paralumbar fossa region. Each temperature logger was suspended close to the peritoneal wall by non‐absorbable suture material (0.4 mm; Vetafil; Bengen, Germany), which was incorporated into the wax coating of the temperature logger and sutured into the muscle layer. The experiment was approved by the Animal Ethics Committee of the University of Western Australia (RA/3/100/875).

#### 2.1.2 Cheetah

Three male and two female wild cheetah (*Acinonyx jubatus*) were implanted with loggers and released at the Tusk Trust Cheetah Rehabilitation Camp (4000 ha) of the AfriCat Foundation (20°50′ S 16°38′ E), in central Namibia during summer. Body mass at temperature logger implantation surgery was 43 ± 6 kg (mean ± SD) (Hetem et al., 2013). Temperature loggers (mlog_T1A, resolution 0.06°C, calibrated accuracy <0.1°C, Sigma Delta Technologies, Australia), were waterproofed with two layers of inert wax and calibrated as described above, dry sterilised using formaldehyde vapour and tethered in the abdominal cavity of the cheetahs via an incision along the linea alba. The temperature loggers were 25 × 25 × 10 mm, weighed approximately 10 g, and measured *T*_c_ every minute. This experiment was approved by the Animal Ethics Screening Committee of the University of the Witwatersrand (2005/42/4).

#### 2.1.3 Mouse

Six (three male, three female) Swiss mice (*Mus musculus*, mean body mass ± SD: 29.7 ± 0.4 g) were housed in individual cages at the Pre‐Clinical Animal Facility at the University of Western Australia, Perth, under a light: dark cycle of 12:12 h, with lights on at 07:00 h. Ambient temperature was maintained at 22.9 ± 0.7°C (mean ± SD). The mice had ad libitum access to standard mouse feed (Specialty Feeds, Glen Forrest, WA, Australia) and drinking water. Mini Mitter transponders (G2 E‐Mitter, 0.1°C resolution, measurement range 33–41°C, Respironics Inc., Pennsylvania, U.S.) were soaked in Actril disinfectant for at least 24 h, and then implanted in the abdominal cavity of mice to measure *T*_c_ at 1‐min intervals. The Mini Mitter transponders communicated with a reading plate (Respironics Inc., Pennsylvania, USA), which was set to transmit *T*_c_ every minute to VitalView software (Respironics Inc., Pennsylvania, USA). The transponders had dimensions of ~15.5 × 6.5 mm, weighed 1.1 g, and were biologically inert and waterproof. The transponders were calibrated pre‐experiment in an insulated water bath (Haake DC30, Germany) against a certified thermometer (Quat 100, Hereaus, Germany, certification by the National Association of Testing Authorities, Australia) to an accuracy of better than 0.1°C. The mouse experiment was approved by the Animal Ethics Committee of the University of Western Australia (RA/3/100/985).

#### 2.1.4 Barnacle goose

Nine wild female adult barnacle geese (*Branta leucopsis*, weighing approximately 1.9 kg) were captured during their post‐breeding wing moult in the Netherlands (51°40′ N, 4°14′ E). Data loggers (DST centi‐HRT, Star‐Oddi, Gardabaer, Iceland) were sterilised and implanted in the abdominal cavity to record *T*_c_ every 5 min (Eichhorn et al., 2021). The loggers were calibrated by the manufacturer, and measured temperature to an accuracy of ±0.2°C at a resolution of 0.032°C. The geese were also harnessed with a solar‐powered tracking device (GsmRadioTag, Milsar Technologies S.R.L.) to aid recovery of temperature loggers. Upon recovery from surgery, the geese were released to the wild at the site where they were captured. The experiment was approved by the Animal Welfare Committees of the Royal Netherlands Academy of Arts and Sciences (licence AVD8010020173788) and the St. Petersburg State University (decision nr. 131‐03‐2 from 3 April 2018).

#### 2.1.5 Pekin duck

Nineteen female Pekin ducks (*Anas platyrhynchos*, body mass ranging 2.7–4.1 kg) were obtained from a boutique commercial free‐range farm in Western Australia. The ducks were group‐housed in outdoor pens, fed once daily with chicken layer pellets and were given ad libitum access to fresh drinking water and open water bathing troughs. Prior to implantation, data loggers (DST micro‐T, resolution 0.06°C, Star‐Oddi, Gardabaer, Iceland) were calibrated in an insulated water bath (Haake DC30, Germany) against a certified mercury‐in‐glass thermometer (WIKA Australia, certified by the National Association of Testing Authorities, Australia) to an accuracy of better than 0.05°C, and sterilised in a chlorhexidine solution for at least 48 h. Data loggers were implanted in the coelomic cavity and recorded *T*_c_ every 5 min (Barrett et al., 2021). The experiment was approved by the Animal Ethics Committee of the University of Western Australia (RA/1/300/1338).

#### 2.1.6 Rabbit

Twenty‐one adult female and 16 adult male wild rabbits (*Oryctolagus cuniculus*, mean body mass at first capture ± SD: 1.6 ± 0.2 kg) were trapped in the field (Vittoria, Central Tablelands, New South Wales, Australia). The animals were inspected for fleas, abnormalities, and clinical signs of myxomatosis. Temperature loggers (mlog_T1A, resolution 0.06°C, calibrated accuracy <0.1°C, Sigma Delta Technologies, Australia) were waterproofed and calibrated as described above for alpaca, and deployed (Maloney et al., 2017). After the temperature loggers were implanted in the abdomen, the rabbits were fitted with radio collars (Sirtrack, Havelock North, New Zealand) to aid recovery of the temperature loggers, and released at the site where they were captured. The work was approved by the New South Wales Department of Primary Industries Animal Ethics Committee (ORA 06/011). The analysed data are from a time when the females were not pregnant.

#### 2.1.7 Rat

Six male Sprague–Dawley rats (*Rattus norvegicus*, body mass between 250 and 300 g) were purchased from the National Health Laboratory Services (Johannesburg, South Africa) and were individually housed in the Central Animal Service unit of the University of the Witwatersrand under a light: dark cycle of 12:12 h, with lights on at 06:00 h. Ambient temperature was maintained at 22 ± 2°C, and rats were given ad libitum access to rodent pellets and water. Abdominal temperature was measured at 1‐min intervals using radiotransmitters (TA10TA‐F40, Data Sciences, St Paul, MN, USA) that were implanted into the peritoneal cavity under deep anaesthesia (Dangarembizi et al., 2017). The radiotransmitters had a resolution of 0.05°C were calibrated by the manufacturer in a water bath to an accuracy of 0.1°C between 35°C and 39°C. The experiment was approved by the Animal Ethics Screening Committee of the University of the Witwatersrand (2014/10/C).

#### 2.1.8 Sheep

Twelve Merino ewes (*Ovis aries*) weighing 49.0 ± 1.1 kg were housed in a large animal facility at the University of Western Australia. To eliminate the effects of ovarian hormones on *T*_c_, the sheep were ovariectomized and given at least 2 weeks to recover from surgery. Abdominal temperature was measured every 5 min using data loggers (Stowaway XTI; Onset Computer, Pocasset, MA) that had a resolution of 0.04°C (Maloney et al., 2013). Prior to abdominal implantation, the loggers were waterproofed with inert wax as described above and calibrated in an insulated water bath (Haake DC30, Germany) against a certified mercury‐in‐glass thermometer (WIKA Australia, certified by the National Association of Testing Authorities, Australia) to an accuracy of better than 0.05°C. Prior to surgical implantation in the abdominal cavity, the loggers were sterilised in a chlorhexidine solution for at least 48 h. Temperature in the large animal facility was maintained between 22–23°C, with a light: dark cycle of 12:12 h and lights on at 07:30 h. Experimental protocols were approved by the Animal Ethics Committee of the University of Western Australia (100/594).

#### 2.1.9 Blue wildebeest

Six female blue wildebeest (*Connochaetes taurinus*, weighing approximately 175 kg) were captured, surgically implanted with temperature transmitters, fitted with a collar that housed a data logger and released on Rooipoort Nature Reserve (42,647 ha, latitude 28°30′–28°40′ S, longitude 24°02′–24°25′ E), located in the Kalahari, approximately 50 km west of Kimberley in the Northern Cape Province of South Africa (Strauss et al., 2016). The temperature transmitters (Africa Wildlife Tracking, Pretoria, South Africa) had a measurement range of 34–50°C and a resolution of 0.03°C. They were calibrated against a high‐accuracy thermometer (Quat 100, Heraeus, Hanau, Germany) in an insulated water bath. Following calibration, the transmitters measured temperature to an accuracy of better than 0.05°C. The transmitters measured abdominal *T*_c_ every 10 min, which was transmitted to and stored on the data logger on the collar. During data collection, the wildebeest lived naturally on the reserve. The experiment was approved by the Animal Ethics Screening Committee of the University of the Witwatersrand (2011/36/05).

### 2.2 Data analysis

A Lomb–Scargle periodogram was applied to the data from each individual of each species to assess whether a rhythm with a period of 24 h was present in the *T*_c_ data. The periodogram analysis was performed in RStudio (RStudio Team, 2021) using R version 4.0.4 (R Core Team, 2021) for Windows. The mesor, amplitude, acrophase and adjusted coefficient of determination that described the daily cosinor rhythm for each individual animal and each sampling interval was calculated in RStudio using the ‘cosinor’ (Sachs, 2014) and ‘card’ (Shah, 2020) packages, as described by the following equation, assuming a period of 24 h (that had been confirmed by the periodogram analysis):

$$Y=M+A\times \cos\left(2\pi \left(t\right)/24+\varphi \right)$$

In the above equation, *M* refers to the midline estimation statistic of rhythm (MESOR), *A* is a variable defining the amplitude, *φ* is a variable describing phase and *t* is time in hours. An adjusted *R* ^2^ was used to prevent a bias in favour of the shorter sampling intervals. Because 5 days of data were used for each individual, the total sample size necessarily decreased as the sampling interval increased.

The cosinor model was applied to each set of re‐sampled data. To mitigate the impact of nonstationarity (i.e., non‐uniformity) of rhythms in longitudinal data on cosinor estimates, 24‐h blocks of data were analysed to generate and plot daily estimates of mesor, amplitude, acrophase and adjusted *R* ^2^ across all of the individuals within each species and the 95% confidence intervals of those data. Because the cosinor variables for individuals within a species were not all altered in the same direction when the data were re‐sampled, the absolute delta changes (Δ) of mesor, amplitude, acrophase and adjusted *R* ^2^, relative to the original sampling interval, were plotted as means ±95% confidence intervals. The number of occurrences when the absolute change exceeded a given threshold was also expressed as a percentage of the total observations per sampling interval per species. Five threshold levels were used for mesor and amplitude (0.1, 0.2, 0.3, 0.4, 0.5°C), acrophase (15 and 30 min, 1, 2, and 3 h), and adjusted *R* ^2^ (0.1, 0.2, 0.3, 0.4, 0.5).

## 3 RESULTS

**FIGURE 2:**
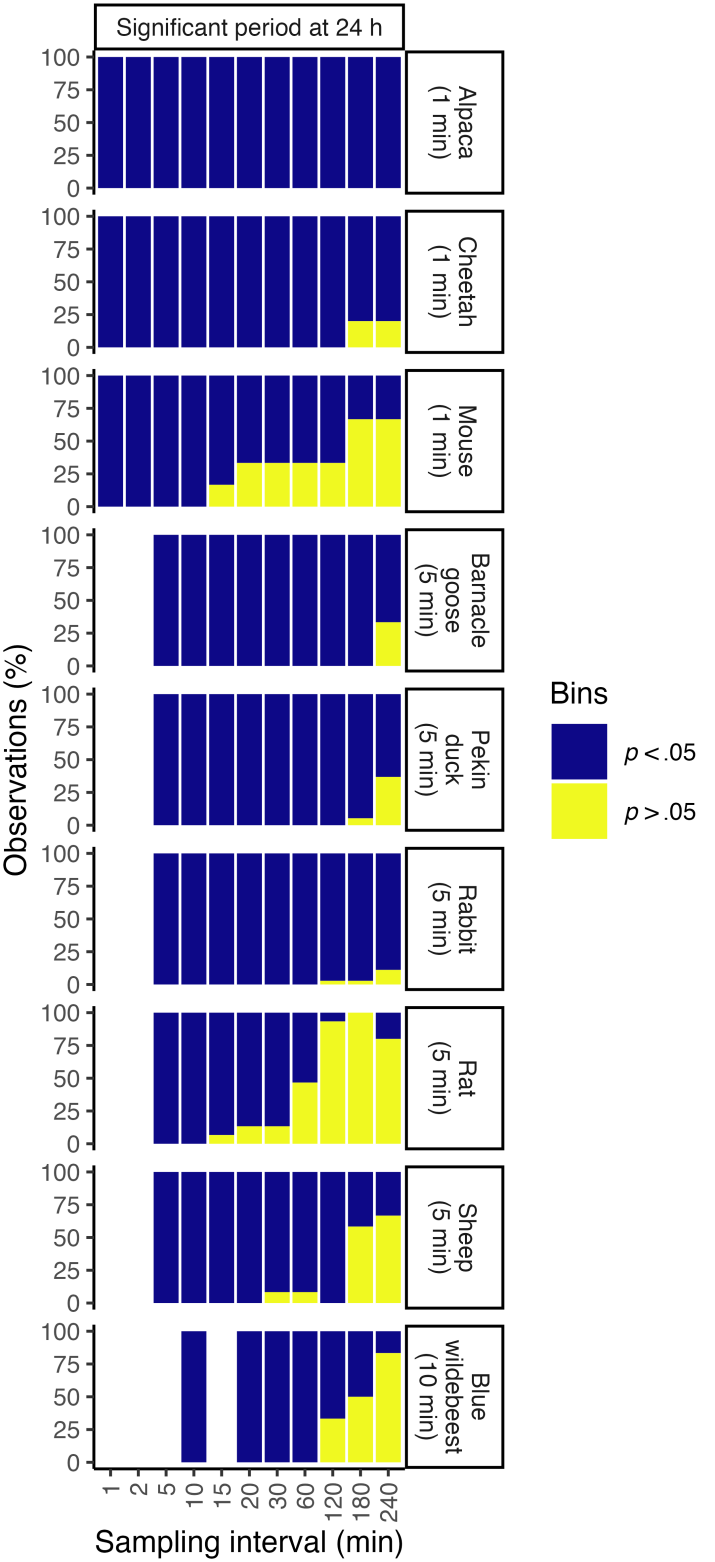
Proportion of individuals of each species that had a significant period at 24 h at each sampling interval The data are derived from the individual periodograms for each species given in Figures A1, A2, A3, A4, A5, A6, A7, A8, A9.

**FIGURE A1:**
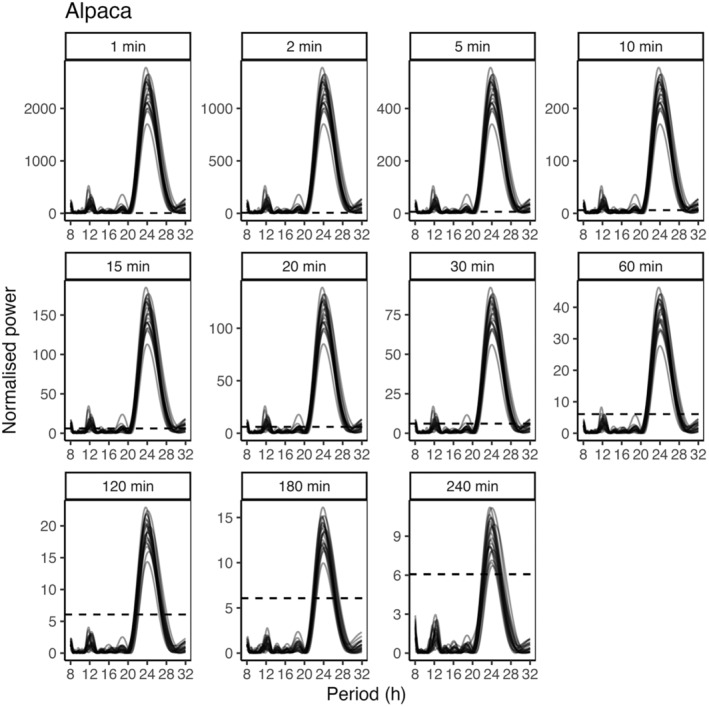
Lomb–Scargle periodogram results for the original and re‐sampled data from alpaca. Individual animals are represented by lines of the same shade in each panel. In every case, the dominant period was at 24 h. Dashed lines on each panel indicate the power level associated with a significant period (*p* < .001). At many of the shorter intervals, the period of 12 h was significant, but at all periods the 24‐h period dominated the periodogram.

**FIGURE A2:**
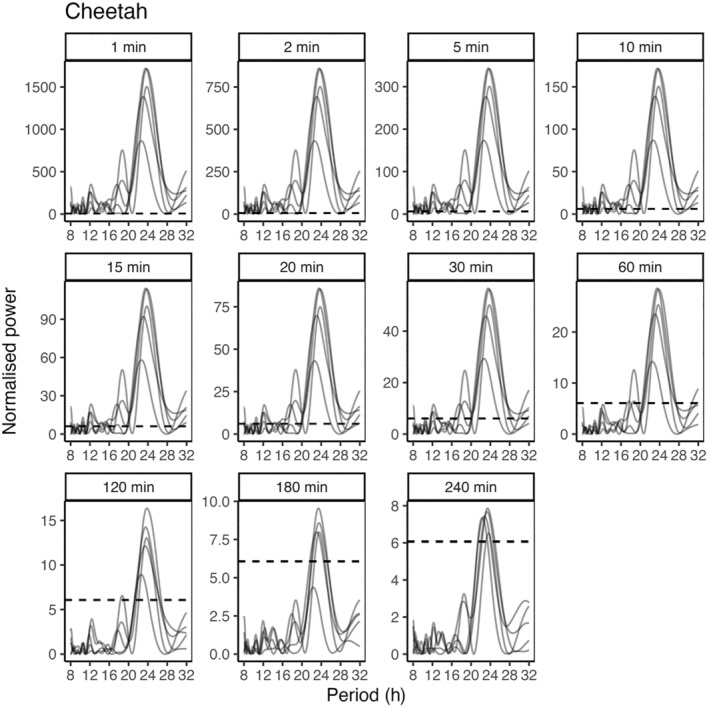
Lomb–Scargle periodogram results for the original and re‐sampled data from cheetah. Individual animals are represented by lines of the same shade in each panel. In every case but one, the dominant period was at 24 h. One individual had a dominant period of about 23 h. Dashed lines on each panel indicate the power level associated with a significant period (*p* < .001). At many of the shorter intervals, periods around 12 h were significant, but at all periods the 24‐h period dominated the periodogram.

**FIGURE A3:**
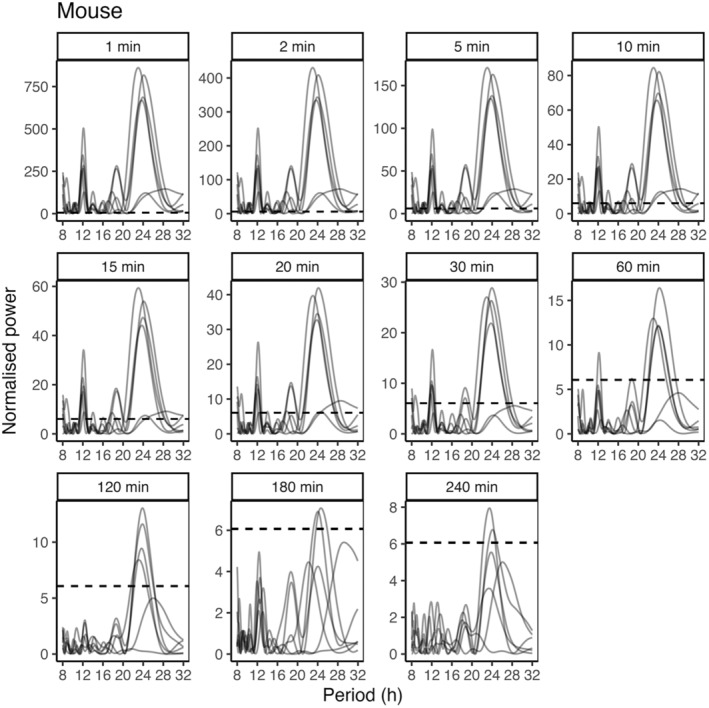
Lomb–Scargle periodogram results for the original and re‐sampled data from mice. Individual animals are represented by lines of the same shade in each panel. In most individuals, the dominant period was at 24 h for all periods, although in two individuals, the dominant period was at 12 h. At many of the shorter intervals, periods around 12 h were significant, but at all periods the 24‐h period dominated the periodogram in all but those two individuals. Dashed lines on each panel indicate the power level associated with a significant period (*p* < .001).

**FIGURE A4:**
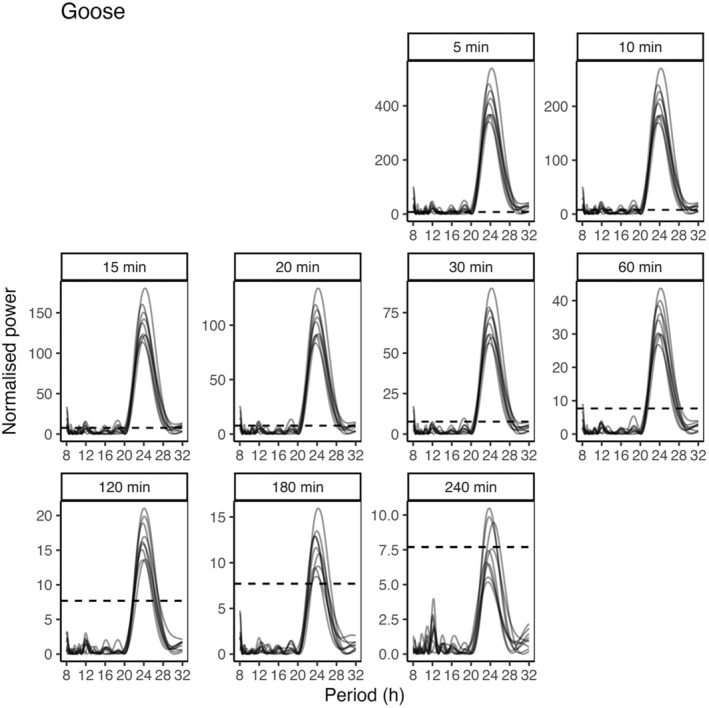
Lomb–Scargle periodogram results for the original and re‐sampled data from geese. Individual animals are represented by lines of the same shade in each panel. In every case, the dominant period was at 24 h. Dashed lines on each panel indicate the power level associated with a significant period (*p* < .001). At many of the shorter intervals, the periods around 12 h were significant, but at all periods the 24‐h period dominated the periodogram.

**FIGURE A5:**
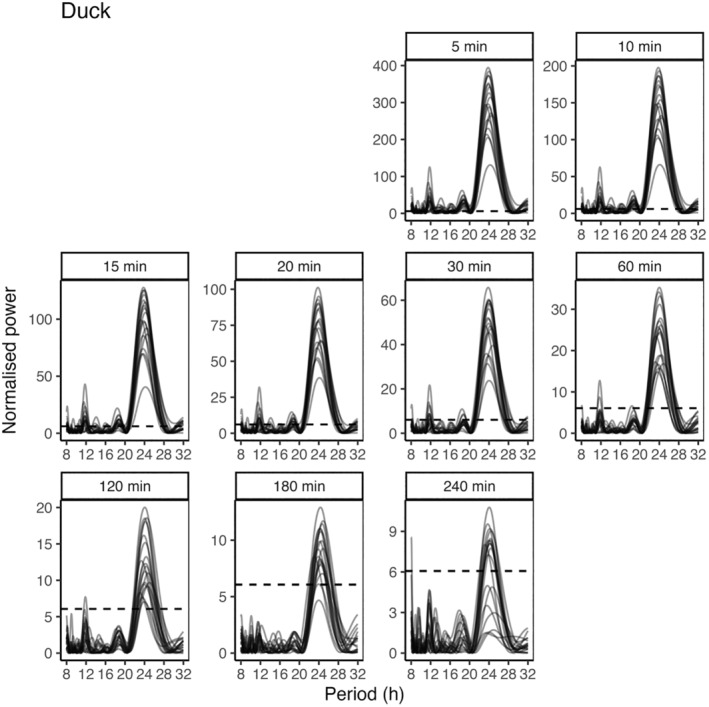
Lomb–Scargle periodogram results for the original and re‐sampled data from ducks. Individual animals are represented by lines of the same shade in each panel. In every case, the dominant period was at 24 h. Dashed lines on each panel indicate the power level associated with a significant period (*p* < .001). At many of the shorter intervals, the periods around 12 h were significant, but at all periods the 24‐h period dominated the periodogram.

**FIGURE A6:**
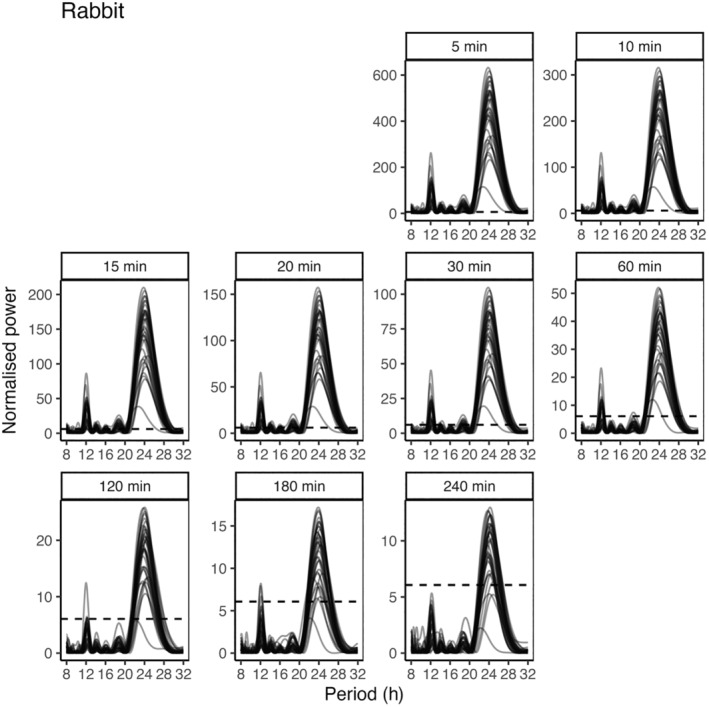
Lomb–Scargle periodogram results for the original and re‐sampled data from rabbits. Individual animals are represented by lines of the same shade in each panel. In every case except one rabbit, the dominant period was at 24 h. That one individual had a dominant period at 12 h, and a secondary peak at around 23 h. Dashed lines on each panel indicate the power level associated with a significant period (*p* < .001). At many of the shorter intervals, the periods around 12 h were significant, but at all periods the 24‐h period dominated the periodogram.

**FIGURE A7:**
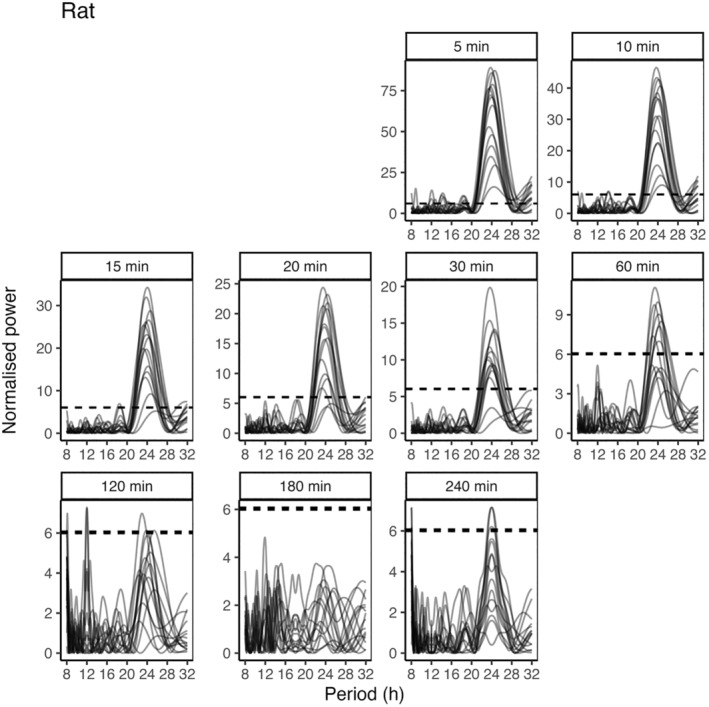
Lomb–Scargle periodogram results for the original and re‐sampled data from rats. Individual animals are represented by lines of the same shade in each panel. In every case, the dominant period was 24 h. Dashed lines on each panel indicate the power level associated with a significant period (*p* < .001). At many of the shorter intervals, the periods around 12 h were significant, but at all periods the 24‐h period dominated the periodogram. At sampling intervals longer than 60 min, the periodograms for most individuals did not reach significance, which would indicate that the animals were aperiodic.

**FIGURE A8:**
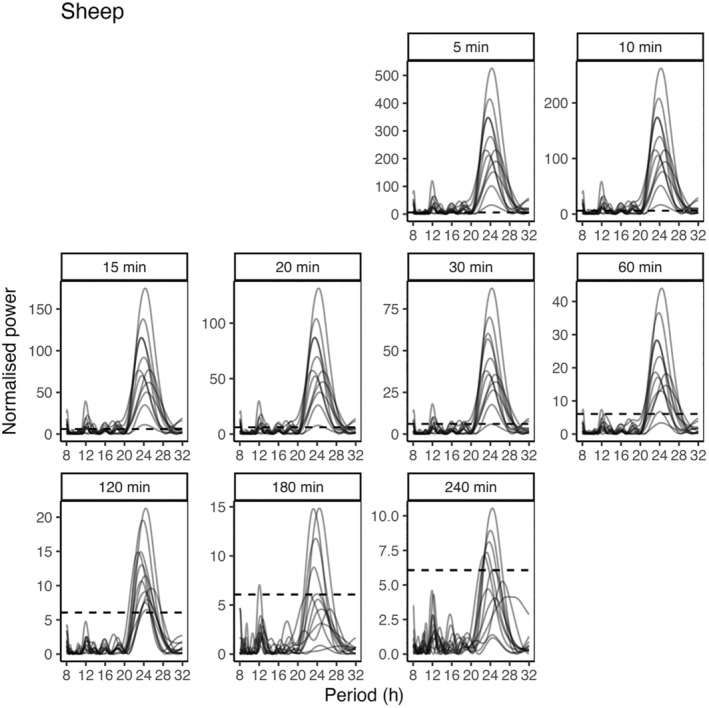
Lomb–Scargle periodogram results for the original and re‐sampled data from sheep. Individual animals are represented by lines of the same shade in each panel. In every case, the dominant period was at 24 h. Dashed lines on each panel indicate the power level associated with a significant period (*p* < .001). At many of the shorter intervals, the periods around 12 h were significant, but at all periods the 24‐h period dominated the periodogram.

**FIGURE A9:**
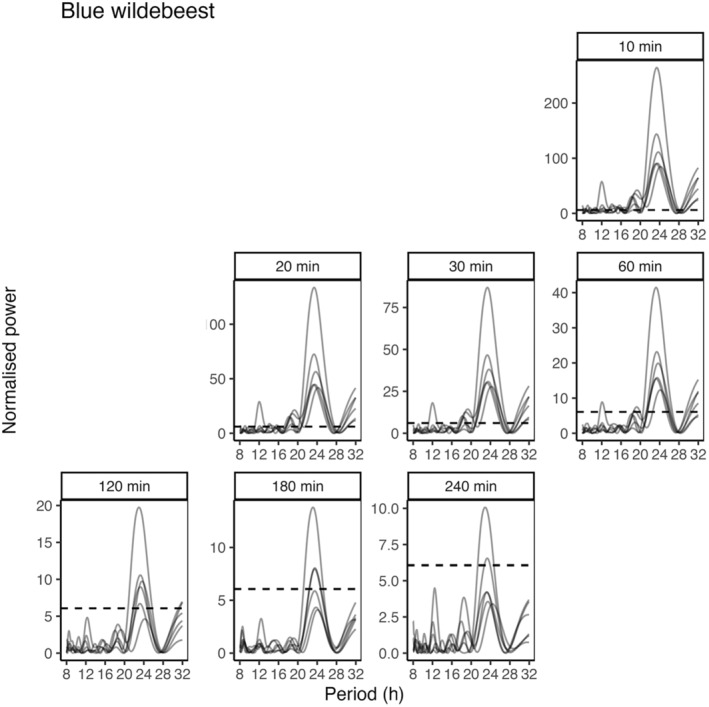
Lomb–Scargle periodogram results for the original and re‐sampled data from blue wildebeest. Individual animals are represented by lines of the same shade in each panel. In every case, the dominant period was at 24 h. Dashed lines on each panel indicate the power level associated with a significant period (*p* < .001). The periodograms for most individuals did not reach significance at periods longer than 120 min.

A significant circadian rhythm with a period of 24 h (Lomb–Scargle periodogram *p* < .001) was detected in each individual in each species at the original sampling interval (Figure 2), although in one rabbit, one sheep and two mice the dominant period was at 12 h, and in one rat, the dominant period was at 32 h. For most individuals, a significant rhythm with a period of 24 h was detected at each re‐sampled interval (Figures A1, A2, A3, A4, A5, A6, A7, A8, A9), although the reliability decreased when the interval was longer than 60 min. In the rat and mouse, the periodogram failed to detect a period at 24 h in most individuals at intervals longer than 120 min (mouse) and 60 min (rat). No rhythm was detected in most individuals of most species at the longest interval of 240 min (Figure 2).

**FIGURE 3:**
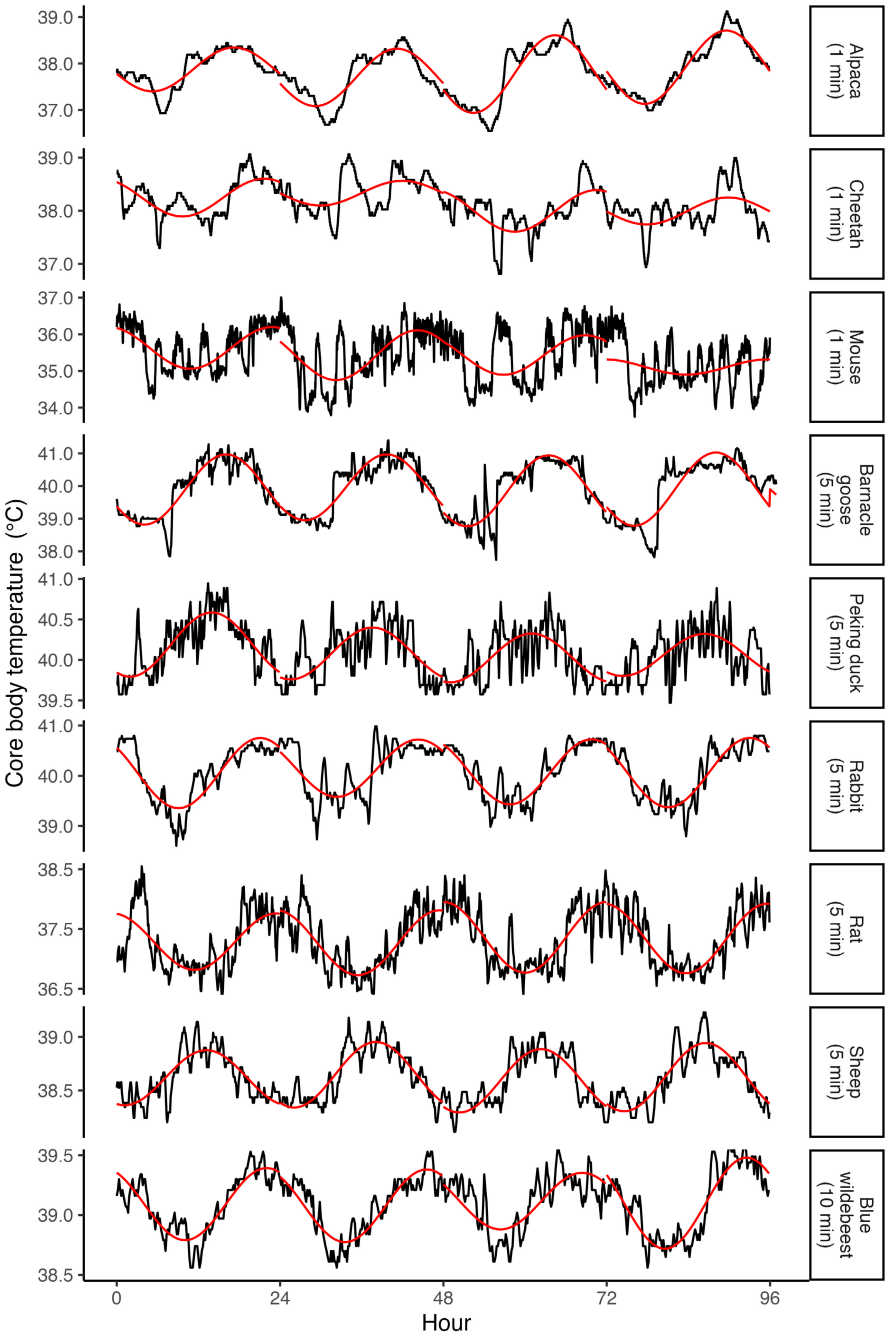
Example *T*_c_ traces (black lines) at the original sampling interval from one individual alpaca, cheetah and mouse (original sampling interval = 1 min), barnacle goose, Pekin duck, rabbit, rat and sheep (original sampling interval = 5 min), and blue wildebeest (original sampling interval = 10 min). The daily fitted cosinor models are shown as a superimposed red trace.

**FIGURE 4:**
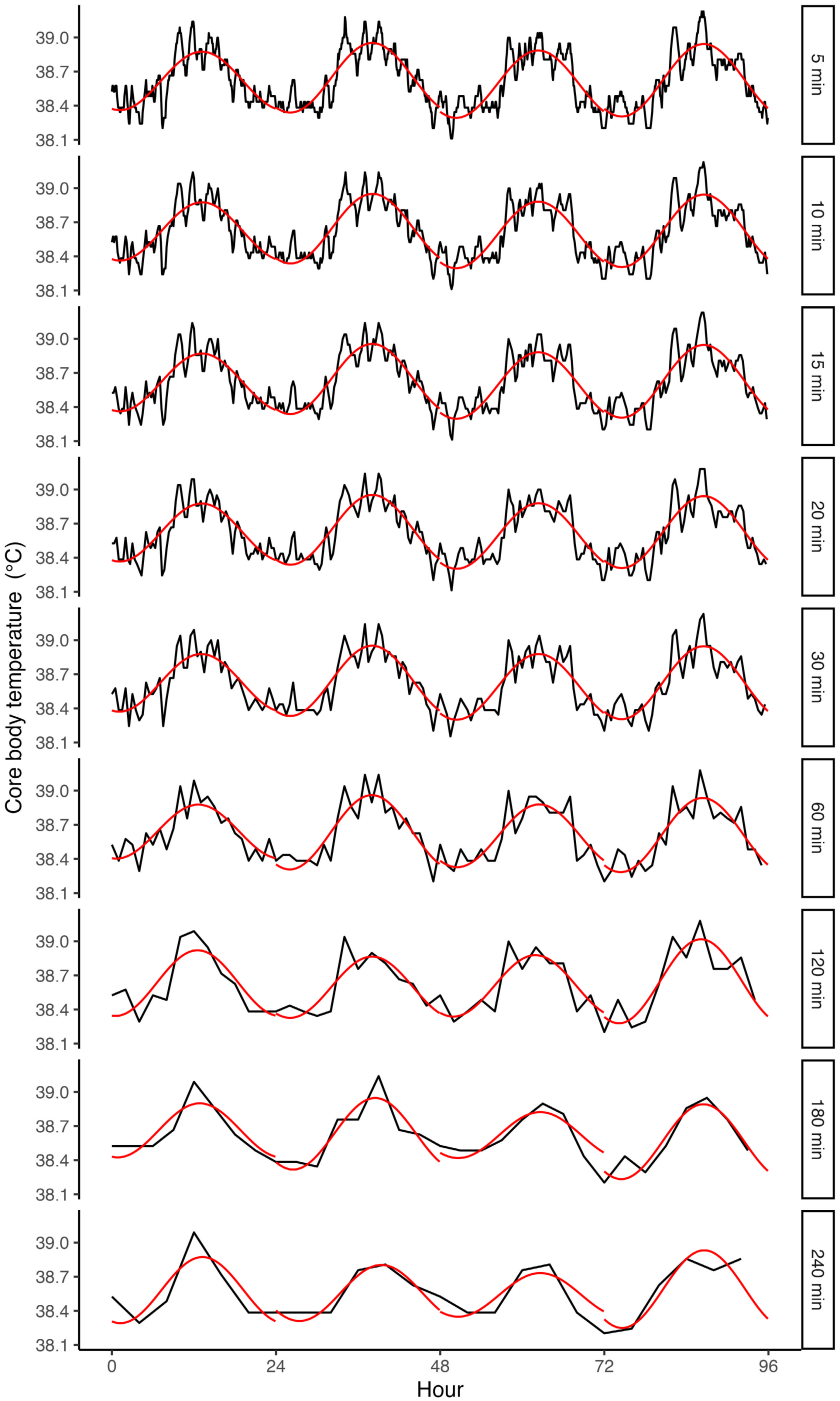
Example *T*_c_ traces from an individual sheep showing raw *T*_c_ data (black) and the daily cosinor model fit (red) for sampling intervals from 1 min to 240 min.

Figure 3 shows representative traces of the original data from one individual of each species, along with the fitted cosine curve for each day. When those data were re‐sampled to simulate longer sampling intervals, the loss of signal resolution led to the elimination of short intra‐day variability in *T*_c_, but the general profile of the circadian rhythm persisted. The impact of resampling at longer intervals is shown for one sheep in Figure 4.

### 3.1 Cosinor analysis

**FIGURE 5:**
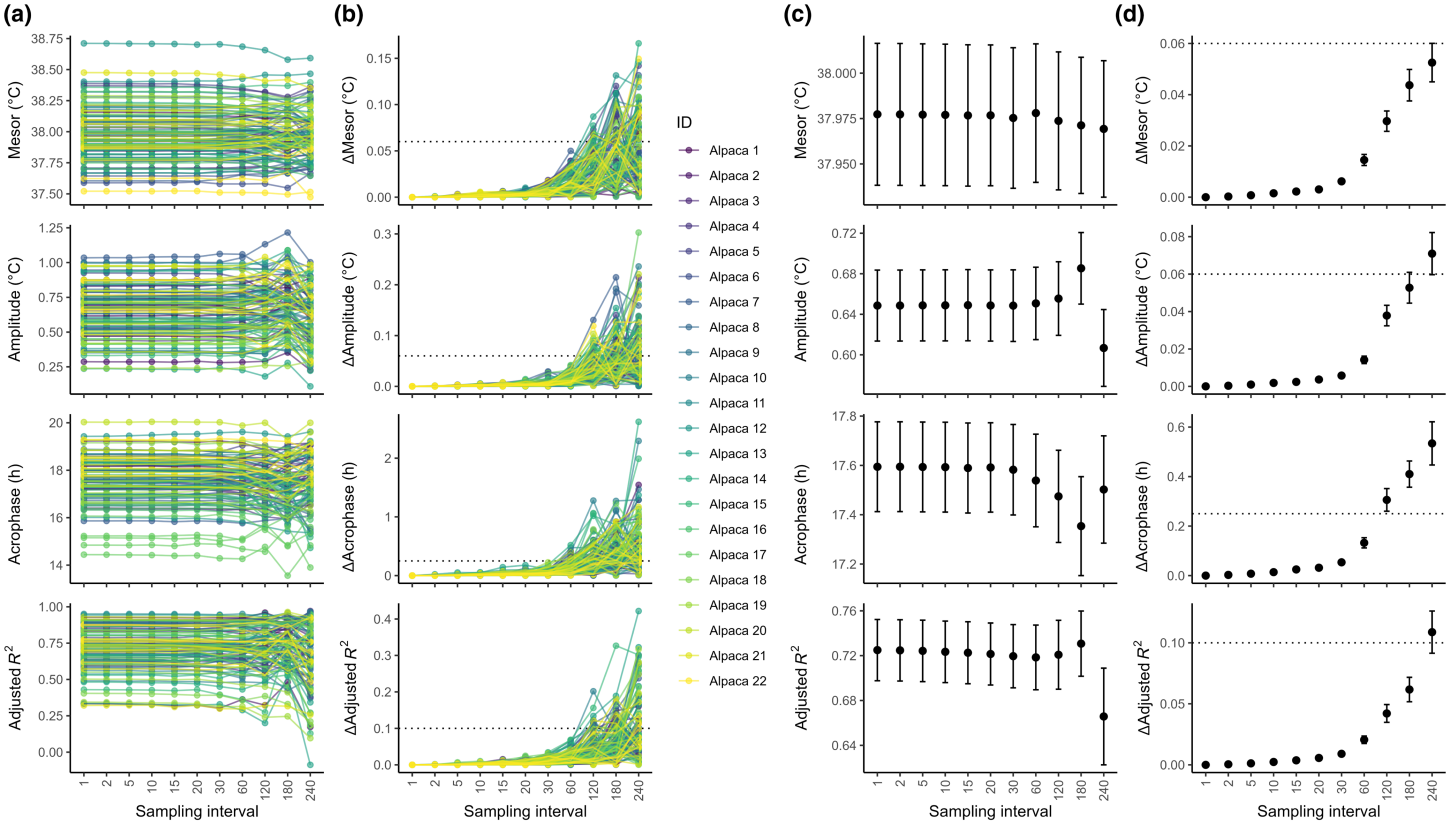
Impact of sampling interval on the mesor, amplitude, acrophase and adjusted *R* ^2^ derived from alpaca *T*_c_ data (5 days × 22 animals). (a) The raw values for each individual and (b) absolute delta change (Δ) from the original sampling interval are shown using the same colour for each individual. (c) The mean change across individuals, and (d) the mean absolute change across individuals for each sampling interval. Panels c and d show mean ± 95% confidence intervals. Horizontal dotted lines indicate Δmesor and Δamplitude = 0.06°C, which is the resolution of the data loggers, and an arbitrary 15‐min threshold for Δacrophase and 0.1 for Δadjusted *R* ^2^.

**FIGURE A10:**
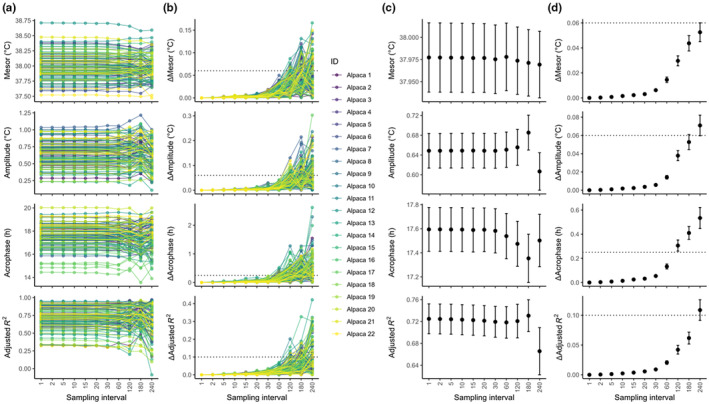
Impact of sampling interval on the mesor, amplitude, acrophase and *R* ^2^ derived from alpaca *T*_c_ data (5 days × 22 animals). (a) The raw values for each individual and (b) absolute delta change (Δ) from the original sampling interval are shown using the same colour for each individual. (c) The mean change across individuals, and (d) the mean absolute change across individuals for each sampling interval. Panels c and d show mean ± 95% confidence intervals. Horizontal dotted lines indicate Δmesor and Δamplitude = 0.06°C, which is the resolution of the data loggers, and an arbitrary 15‐min threshold for Δacrophase and 0.1 for Δadjusted *R* ^2^.

**FIGURE A11:**
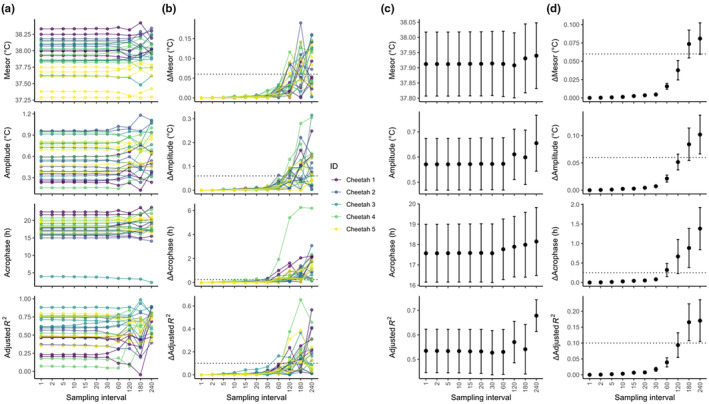
Impact of sampling interval on the mesor, amplitude, acrophase and *R* ^2^ derived from cheetah *T*_c_ data (5 days × 5 animals). (a) The raw values for each individual and (b) absolute delta change (Δ) from the original sampling interval are shown using the same colour for each individual. (c) The mean change across individuals, and (d) the mean absolute change across individuals for each sampling interval. Panels c and d show mean ± 95% confidence intervals. Horizontal dotted lines indicate Δmesor and Δamplitude = 0.06°C, which is the resolution of the data loggers, and an arbitrary 15‐min threshold for Δacrophase and 0.1 for Δadjusted *R* ^2^.

**FIGURE A12:**
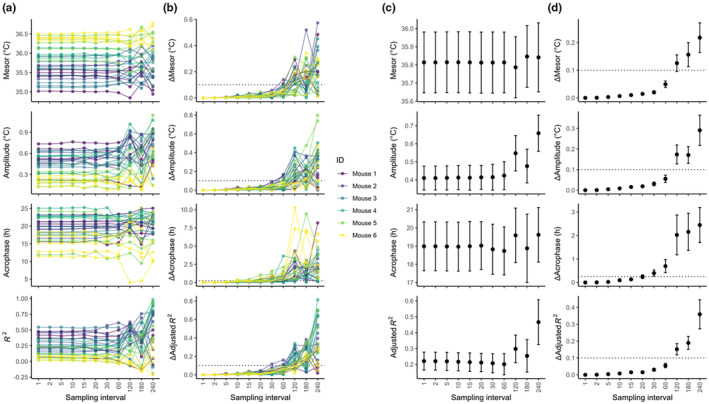
Impact of sampling interval on the mesor, amplitude, acrophase and *R* ^2^ derived from mouse *T*_c_ data (5 days × 6 animals). (a) The raw values for each individual and (b) absolute delta change (Δ) from the original sampling interval are shown using the same colour for each individual. (c) The mean change across individuals, and (d) the mean absolute change across individuals for each sampling interval. Panels c and d show mean ± 95% confidence intervals. Horizontal dotted lines indicate Δmesor and Δamplitude = 0.1°C, which is the resolution of the data transponders, and an arbitrary 15‐min threshold for Δacrophase and 0.1 for Δadjusted *R* ^2^.

**FIGURE A13:**
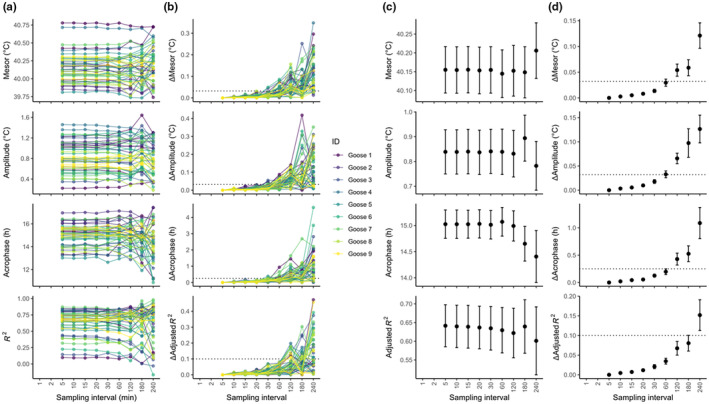
The impact of sampling interval on the mesor, amplitude, acrophase, and *R* ^2^ derived from barnacle goose *T*_c_ data (5 days × 9 animals). (a) The raw values for each individual and (b) absolute delta change (Δ) from the original sampling interval are shown using the same colour for each individual. (c) The mean change across individuals, and (d) the mean absolute change across individuals for each sampling interval. Panels c and d show mean ± 95% confidence intervals. Horizontal dotted lines indicate Δmesor and Δamplitude = 0.032°C, which is the resolution of the data loggers, and an arbitrary 15‐min threshold for Δacrophase and 0.1 for Δadjusted *R* ^2^.

**FIGURE A14:**
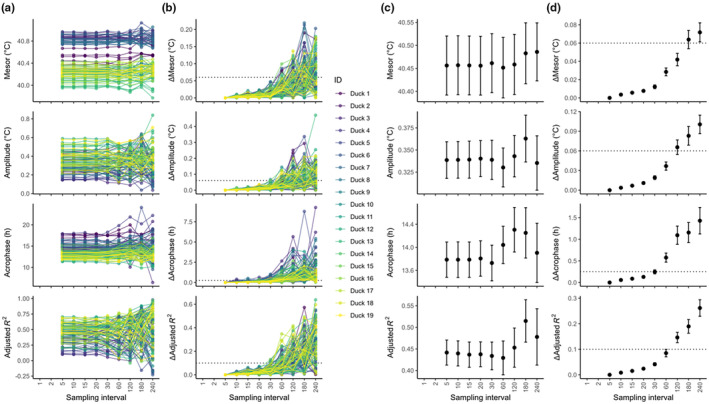
Impact of sampling interval on the mesor, amplitude, acrophase and *R* ^2^ derived from Pekin duck *T*_c_ data (5 days × 19 animals). (a) The raw values for each individual and (b) absolute delta change (Δ) from the original sampling interval are shown using the same colour for each individual. (c) The mean change across individuals, and (d) the mean absolute change across individuals for each sampling interval. Panels c and d show mean ± 95% confidence intervals. Horizontal dotted lines indicate Δmesor and Δamplitude = 0.06°C, which is the resolution of the data loggers, and an arbitrary 15‐min threshold for Δacrophase and 0.1 for Δadjusted *R* ^2^.

**FIGURE A15:**
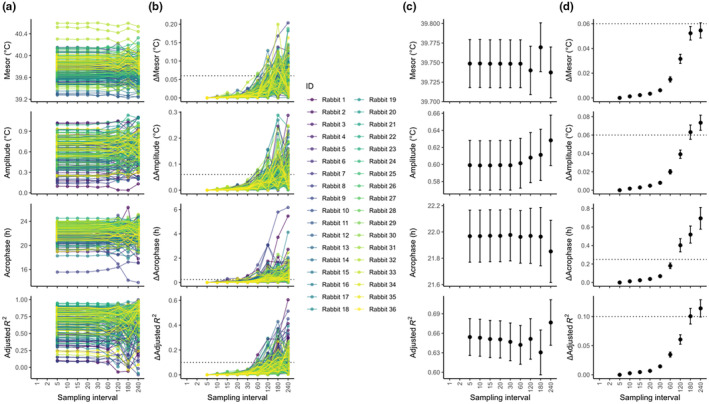
Impact of sampling interval on the mesor, amplitude, acrophase and *R* ^2^ derived from rabbit *T*_c_ data (5 days × 36 animals). (a) The raw values for each individual and (b) absolute delta change (Δ) from the original sampling interval are shown using the same colour for each individual. (c) The mean change across individuals, and (d) the mean absolute change across individuals for each sampling interval. Panels c and d show mean ± 95% confidence intervals. Horizontal dotted lines indicate Δmesor and Δamplitude = 0.06°C, which is the resolution of the data loggers, and an arbitrary 15‐min threshold for Δacrophase and 0.1 for Δadjusted *R* ^2^.

**FIGURE A16:**
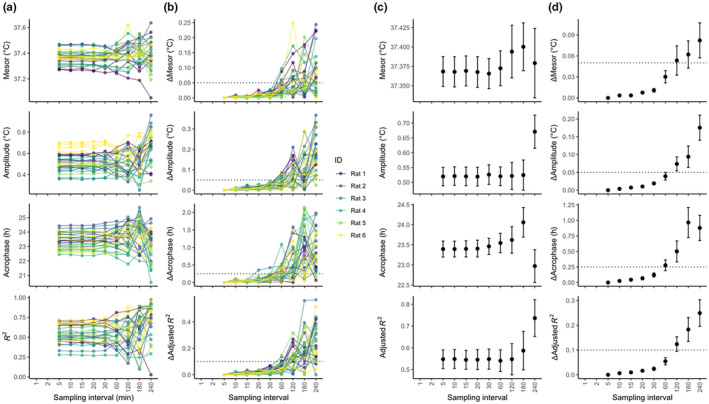
Impact of sampling interval on the mesor, amplitude, acrophase and *R* ^2^ derived from rat *T*_c_ data (5 days × 6 animals). (a) The raw values for each individual and (b) absolute delta change (Δ) from the original sampling interval are shown using the same colour for each individual. (c) The mean change across individuals, and (d) the mean absolute change across individuals for each sampling interval. Panels c and d show mean ± 95% confidence intervals. Horizontal dotted lines indicate Δmesor and Δamplitude = 0.05°C, which is the resolution of the data transponders, and an arbitrary 15‐min threshold for Δacrophase and 0.1 for Δadjusted *R* ^2^.

**FIGURE A17:**
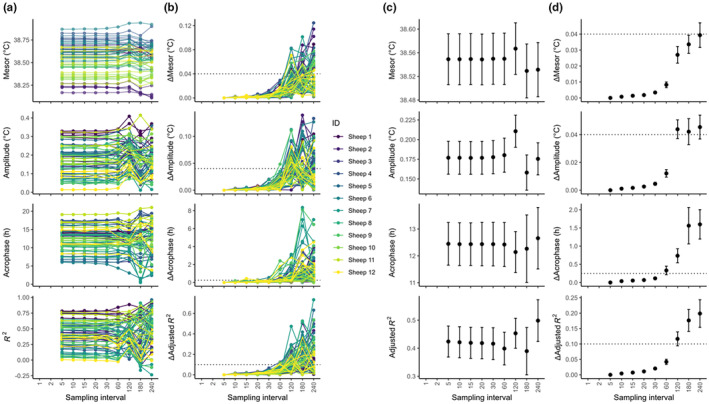
Impact of sampling interval on the mesor, amplitude, acrophase, and *R* ^2^ derived from sheep *T*_c_ data (5 days × 12 animals). (a) The raw values for each individual and (b) absolute delta change (Δ) from the original sampling interval are shown using the same colour for each individual. (c) The mean change across individuals, and (d) the mean absolute change across individuals for each sampling interval. Panels c and d show mean ± 95% confidence intervals. Horizontal dotted lines indicate Δmesor and Δamplitude = 0.04°C, which is the resolution of the data loggers, and an arbitrary 15‐min threshold for Δacrophase and 0.1 for Δadjusted *R* ^2^.

**FIGURE A18:**
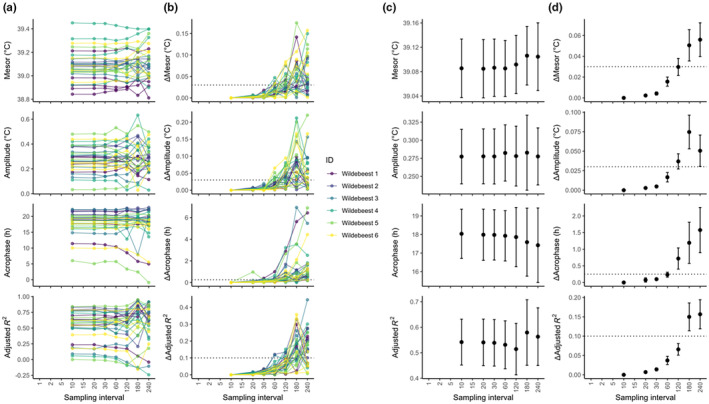
Impact of sampling interval on the mesor, amplitude, acrophase and *R* ^2^ derived from blue wildebeest *T*_c_ data (5 days × 6 animals). (a) The raw values for each individual and (b) absolute delta change (Δ) from the original sampling interval are shown using the same colour for each individual. (c) The mean change across individuals, and (d) the mean absolute change across individuals for each sampling interval. Panels c and d show mean ± 95% confidence intervals. Horizontal dotted lines indicate Δmesor and Δamplitude = 0.04°C, which is the resolution of the data loggers, and an arbitrary 15‐min threshold for Δacrophase and 0.1 for Δadjusted *R* ^2^.

In all species, there was a large individual variability in all of the characteristics of the rhythm obtained from cosinor analysis at the different sampling intervals (Figure 5, panels a and c for the alpaca, Figures A10, A11, A12, A13, A14, A15, A16, A17, A18, panels a and c for all nine species). In each individual profile in each species, the 95% confidence intervals show that the estimates of the cosinor *T*_c_ rhythm began to deviate from the original estimates at all re‐sampled intervals, but the absolute change of the mesor and amplitude generally stayed within the resolution of the loggers at the 60‐min sampling interval (Figure 5, panels b and d for the alpaca, Figures A10, A11, A12, A13, A14, A15, A16, A17, A18, panels b and d for all nine species). The amplitude and adjusted *R* ^2^ tended to increase as the sampling interval increased, probably owing to the exclusion of ultradian fluctuations in *T*_c_, but the direction of change in mesor and acrophase did not appear to follow a general trend across species. A summary of the results for each species is given below.

#### 3.1.1 Alpaca

There was no significant change from the original data in the mesor, amplitude, acrophase or adjusted *R* ^2^ at any sampling interval (Figure 5). The Δmesor and Δamplitude remained within the logger resolution, even up to 240 min (Δmesor, 95% CI [0.045, 0.060]; Δamplitude, [0.060, 0.082]). The Δacrophase exceeded 15 min of the original sampling interval at the 120‐min interval (95% CI [0.260, 0.352]) and longer, and the Δadjusted *R* ^2^ approached 0.1 of the original sampling interval when re‐sampled at a 240‐min interval (95% CI [0.091, 0.126]).

#### 3.1.2 Cheetah

There was no significant change from the original data in the mesor, amplitude, acrophase or adjusted *R* ^2^ at any sampling interval (Figure A11). The Δmesor and Δamplitude both approached the resolution of the data logger when re‐sampled at 120‐min intervals (Δmesor, 95% CI [0.054, 0.093]; Δamplitude, [0.054, 0.113]), and exceeded it at a 240‐min interval (Δmesor, 95% CI [0.060, 0.102]; Δamplitude, [0.066, 0.137]). When re‐sampled at 60‐min intervals, the Δacrophase trended towards exceeding 15 min (95% CI [0.152, 0.491]), and exceeded it when re‐sampled at 180‐min intervals and above (95% CI [0.229, 1.105]). At 180‐min intervals and longer, the Δadjusted *R* ^2^ exceeded 0.1 (95% CI [0.108, 0.223]) of the original sampling interval.

#### 3.1.3 Mouse

There was no significant change from the original data in the mesor or acrophase at any sampling interval (Figure A12). The amplitude and adjusted *R* ^2^ were increased at the 240‐min sampling interval compared with the original sampling interval (amplitude: 1‐min 95% CI [0.343, 0.476], 240‐min [0.558, 0.758]; adjusted *R* ^2^: 1‐min, [0.164, 0.277], 240‐min, [0.325, 0.607]). The Δmesor approached the resolution of the data loggers at 120‐min intervals (95% CI [0.095, 0.155]), and both Δmesor and Δamplitude exceeded it at 120‐min intervals and above (95% CI [0.113, 0.199]) and [0.128, 0.219], respectively. At sampling intervals longer than 30 min, the Δacrophase exceeded 15 min (95% CI [0.266, 0.533]), and Δadjusted *R* ^2^ exceeded 0.1 at sampling intervals longer than 120 min (95% CI [0.118, 0.185]).

#### 3.1.4 Barnacle goose

There was no significant change from the original data in the mesor, amplitude, acrophase or adjusted *R* ^2^ at any sampling interval (Figure A13). At 120‐min intervals and above, the Δmesor and Δamplitude both exceeded the resolution of the data logger (Δmesor: 95% CI [0.042, 0.066]; Δamplitude, [0.055, 0.077]), and the Δacrophase (95% CI [0.320, 0.537]) exceeded 15 min of the original sampling interval. The Δadjusted *R* ^2^ remained within 0.1 of the original sampling interval when re‐sampled, except at 240‐min intervals (95% CI [0.112, 0.191]).

#### 3.1.5 Pekin duck

There was no significant change from the original data in the mesor, amplitude, acrophase or adjusted *R* ^2^ at any sampling interval (Figure A14). The Δmesor exceeded the resolution of the data loggers when re‐sampled at intervals of 180 min (95% CI [0.053, 0.074]) and 240 min (95% CI [0.061, 0.082]). Likewise, the Δamplitude approached and exceeded the resolution of the data loggers at 120 (95% CI [0.054, 0.077]) and 180 (95% CI [0.069, 0.097]) min intervals and above. The Δacrophase exceeded 15 min of the original sampling interval (95% CI [0.470, 0.682]) at the 60‐min interval and above, and Δadjusted *R* ^2^ exceeded 0.1 (95% CI [0.0126, 0.166]) of the original sampling interval at the 120‐min interval and above.

#### 3.1.6 Rabbit

There was no significant change from the original data in the mesor, amplitude, acrophase or adjusted *R* ^2^ at any sampling interval (Figure A15). When re‐sampled at 240‐min intervals, Δmesor approached the resolution of the data logger (95% CI [0.048, 0.061]), while Δamplitude exceeded it (95% CI [0.065, 0.082]). The Δacrophase exceeded 15 min of the original sampling interval when re‐sampled at the 120‐min interval (95% CI [0.332, 0.474]) and longer, and the Δadjusted *R* ^2^ trended towards exceeding 0.1 of the original sampling interval at the 240‐min interval (95% CI [0.099, 0.128]).

#### 3.1.7 Rat

There was no significant change from the original data in the mesor or acrophase at any sampling interval (Figure A16). The amplitude and adjusted *R* ^2^ were increased at the 240‐min sampling interval compared with the original sampling interval (amplitude: 1‐min 95% CI [0.488, 0.551], 240‐min [0.615, 0.727]; adjusted *R* ^2^: 1‐min, [0.505, 0.590], 240‐min, [0.652, 0.821]). The Δmesor and Δamplitude exceeded the resolution of the data loggers when re‐sampled at 240‐min intervals (95% CI [0.057, 0.107]) and at 120‐min intervals (95% CI [0.054, 0.093]) and above. The Δacrophase exceeded 15 min of the original sampling interval when re‐sampled at 120‐min intervals (95% CI [0.332, 0.670]) and above. The adjusted Δ*R* ^2^ approached exceeding 0.1 of the original sampling interval when re‐sampled at a 120‐min interval (95% CI [0.094, 0.153]), and exceeded it at a 180‐min interval and above (95% CI [0.135, 0.231]).

#### 3.1.8 Sheep

There was no significant change from the original data in the mesor, amplitude, acrophase or adjusted *R* ^2^ at any sampling interval (Figure A17). The Δmesor and Δamplitude were within the logger resolution, even when re‐sampled at 240‐min intervals (Δmesor, 95% CI [0.032, 0.047]; Δamplitude, [0.037, 0.054]). The Δacrophase trended towards exceeding 15 min of the original sampling interval when re‐sampled at 60‐min intervals (95% CI [0.214, 0.448]) and exceeded it when re‐sampled at 120‐min intervals and above (95% CI [0.542, 0.929]). The Δadjusted *R* ^2^ trended towards exceeding 0.1 of the original sampling interval when re‐sampled at 120‐min intervals (95% CI [0.094, 0.139]), and exceeded it at 180‐min intervals and above (95% CI [0.141, 0.212]).

#### 3.1.9 Blue wildebeest

There was no significant change from the original data in the mesor, amplitude, or acrophase at any sampling interval (Figure A18). The Δmesor and Δamplitude both exceeded the resolution of the data loggers when re‐sampled at 180‐min intervals and above (Δmesor, 95% [0.035, 0.066]), (Δamplitude [0.053, 0.096]), with Δamplitude at 120‐min interval also trending towards exceeding the resolution of the data loggers (95% CI [0.027, 0.047]). The Δacrophase exceeded 15 min of the original sampling interval at 120‐min intervals (95% CI [0.402, 1.038]) and longer, and the Δadjusted *R* ^2^ exceeded 0.1 of the original sampling interval at 180‐min intervals (95% CI [0.114, 0.186]) and longer.

### 3.2 Proportion of results that deviated from the original estimates

**FIGURE 6:**
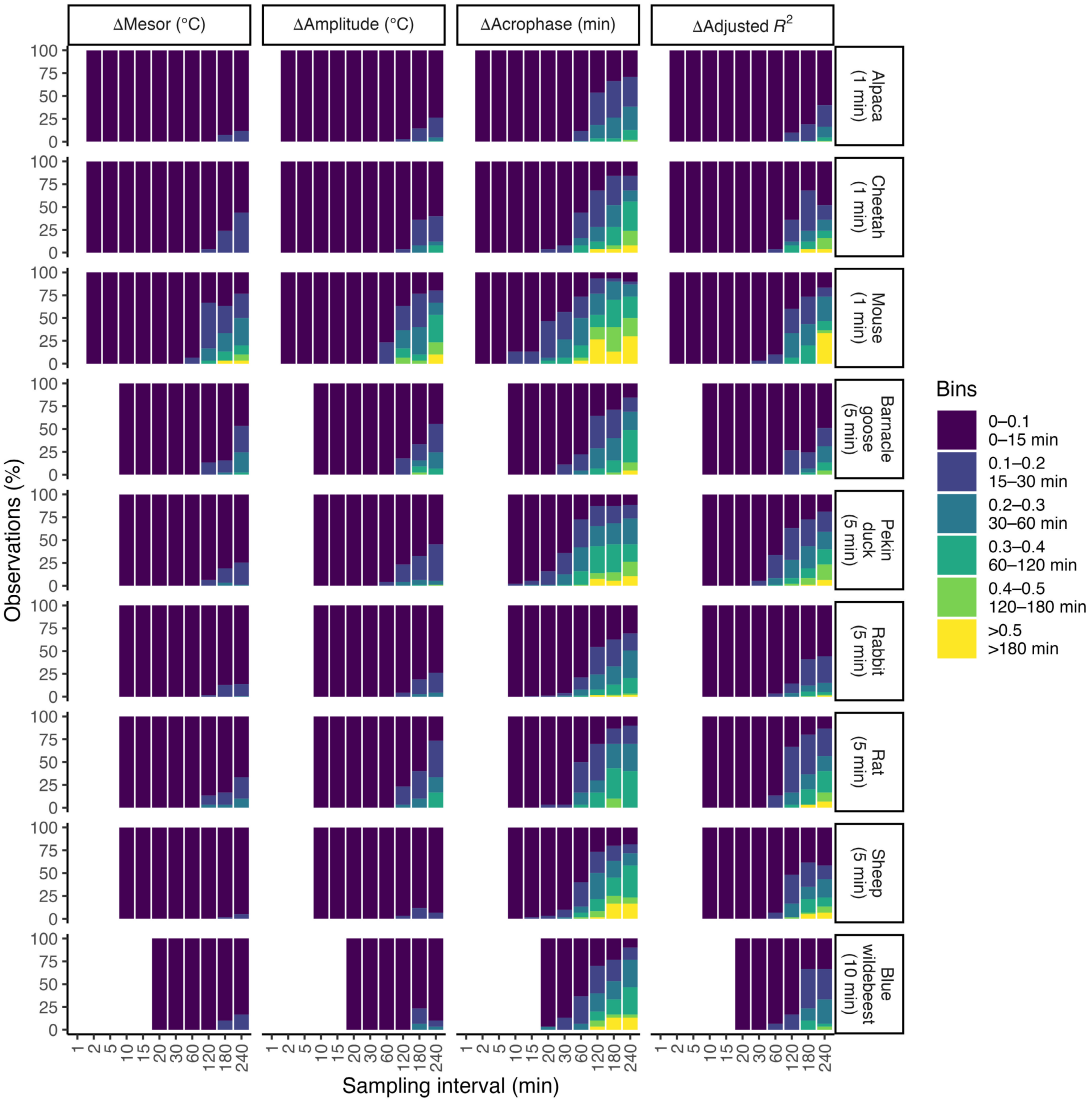
Deviation of cosinor variables at each interval from estimates made from data at the original interval. The proportion of estimates of each cosinor variable that deviated by given amounts from the value derived from the original data are indicated by the colour scale bar. The proportion increased as the sampling interval increased for all species (alpaca, *n* = 22; cheetah, *n* = 5; mouse, *n* = 6; barnacle goose, *n* = 9; Pekin duck, *n* = 19; rabbit, *n* = 36; rat, *n* = 6; sheep, *n* = 12; blue wildebeest, *n* = 6).

The direction of deviation of individuals from the original cosinor estimates within each species was inconsistent, which suggests that merely comparing the averaged cosinor estimates of the re‐sampled data to the original data may not necessarily be a reliable indicator of how well the re‐sampled data represent the original data (Figure 5b for the alpaca, Figures A11, A12, A13, A14, A15, A16, A17, A18 for all species). An alternative method of measuring the impact of sampling interval on the estimate of the cosinor *T*_c_ rhythm is to quantify the proportion of results at each sampling interval that deviate, in either direction, from the cosinor estimates derived from the original sampling interval (Figure 5b for the alpaca, Figures A11, A12, A13, A14, A15, A16, A17, A18 for all species) and to sum those individuals that deviated at each interval. Overall, all of the estimates of the cosinor variables remained very consistent up to about the 60‐min interval (Figure 6). Details for each characteristic of the cosinor *T*_c_ rhythm are given below.

#### 3.2.1 Mesor

The mesor showed the greatest tolerance to changes in the sampling interval. More than 90% of observations in all species deviated by less than 0.1°C at up to a 60‐min sampling interval. Even when the sampling interval was 240 min, fewer than half of the observations of Δmesor showed a deviation of more than 0.1°C. The exceptions were the mouse and the goose, where 77% and 53% of the data, respectively, deviated by more than 0.1°C.

#### 3.2.2 Amplitude

No estimate of amplitude in any species differed by more than 0.1°C from the original sampling interval when data were re‐sampled at up to 30‐min intervals. At longer intervals, the proportion of observations where Δamplitude exceeded 0.1°C progressively increased as the sampling interval increased, with the mouse (80% of Δamplitude >0.1°C) and the rat (63% of Δamplitude >0.1°C) showing the largest Δamplitude when the data were re‐sampled at a 240‐min interval, and when some estimates of the amplitude exceeded the original by 0.5°C.

#### 3.2.3 Acrophase

Of all the cosinor variables, acrophase appeared to be the most sensitive to the sampling interval. Observations when Δacrophase differed from the original value by 15 min first occurred when data were re‐sampled at a 10‐min interval in the mouse (13%) and the duck (2%). Despite that generality, the Δacrophase remained less than 15 min different from the original at a 30‐min sampling interval in the alpaca (100%), cheetah (92%), sheep (90%), rabbit (96%), blue wildebeest (87%) and goose (89%), but differed by more than 60 min in the mouse (26%), duck (12%), and rat (14%) at the same interval. At higher sampling intervals, shifts in acrophase of more than 180 min were observed in all species except the alpaca.

#### 3.2.4 Adjusted *R* ^2^

The proportion of the circadian rhythm that was explained by the cosinor model (the adjusted *R* ^2^) remained within ±0.1 of the original sampling interval up to 30 min for all species, except for 3% for the mouse and 5% for the duck that were within ±0.2. At a 60‐min sampling interval, the adjusted *R* ^2^ remained within ±0.1 for >90% of all individuals within a species, except the rat (87%) and duck (66%). When data were re‐sampled to 240 min, adjusted Δ*R* ^2^ of up to 0.5 were observed in data from all species except the alpaca, goose and wildebeest, with the highest proportion in the mouse.

## 4 DISCUSSION

Our study establishes that near‐continuous sampling of *T*_c_ is unnecessary for reliable estimation of the circadian rhythm of *T*_c_ using periodogram and cosinor analysis but that longer sampling intervals have varying effects on the accuracy of estimation, depending on species and the circadian variable in question. In general, estimates of the period of the rhythm were reliable in the larger species at up to 120‐min intervals, but failed to detect a rhythm in the smaller species at 60‐min intervals. Estimates of mesor remained accurate even at sampling intervals longer than 2 h, while estimates of acrophase exhibited shifts of up to 30 min when the interval was 60 min. Estimates of amplitude and the strength of the fit (*R* ^2^) tended to remain accurate up to a 60‐min sampling interval. Although there were some statistically significant changes in mesor and amplitude at sampling intervals of 60 min or shorter, the differences were smaller than the resolution of most temperature loggers and so are probably not biologically significant. Based on these results, we propose that a sampling frequency of 30 min offers a satisfactory balance between sufficient signal resolution for the accurate estimation of period and cosinor variables and the maximisation of deployment time. For investigations where the precise estimation of acrophase is not necessary, sampling intervals may be extended up to 60 min. Nevertheless, caution should be applied when investigating animals that have large ultradian *T*_c_ fluctuations, such as mice, or species for which body temperature patterns are unknown.

In its simplest interpretation, the Nyquist–Shannon criteria states that a digital sampling rate must be at least twice the highest frequency contained within the signal of interest (Shannon, 1949). If the rhythm of core body temperature is truly circadian with a period of 24 h, and the Nyquist–Shannon criteria applies to the analysis of that rhythm, then sampling at just over twice per day should provide for a reasonable estimate of the period of the rhythm. We can confirm the first of those two statements because periodogram analysis of the original data revealed a dominant, and significant, period at 24 h for the vast majority of individuals of all nine species. One rabbit, one sheep and two mice had a dominant period at 12 h, and one rat had a dominant period at 32 h. We have no explanation for those individual differences. However, our analyses did not support the contention that thrice‐daily sampling would resolve the period. For most individuals, the longer periods that we analysed failed to reach significance for many individuals, and our longest period was 240 min, providing six samples per day. For the rat and the mouse, estimates of the period were reliable up to only 15‐min intervals, and for the other species, the analysis became unreliable above about 120‐min intervals. We conclude that the Nyquist–Shannon criteria cannot be applied to adequately determine the period of the body temperature rhythm in mammals and birds, and that sampling must be at least six times per day for most species, and much more frequent for smaller species such as the rat and the mouse. Because we analysed 5 days per individual, it is possible that these estimates would be different when more days are analysed.

Estimation of the amplitude of a rhythm can be more problematic because of signal aliasing, when the sampling rate is too low to detect the actual rhythm. In the case of aliasing, the temporal relation between the acrophase of a signal and the timing of each sample becomes critical (Glaser & Ruchkin, 1976). For example, if a temperature rhythm that fits a sinusoid peaks and troughs at midday and midnight, respectively, and we sample at midday and midnight each day, we could reliably detect the rhythm and the amplitude. But if we sampled at 06:00 h and 18:00 h each day, we would obtain the same reading at each sample, and conclude that there is no rhythm, irrespective of what is the actual amplitude of the rhythm. Thus, a second general rule of waveform analysis is that to adequately resolve the amplitude, a given analogue waveform must be sampled at least three to five times per cycle (Glaser & Ruchkin, 1976). The longest period that we tested was 4 h, providing for six samples per day. Even at six samples per day, the estimates of amplitude and acrophase were unreliable, if we take the original sampling interval as providing the true estimate. Even more frequent sampling at 24 samples per day (1‐h intervals) led to errors in the estimation of the amplitude and acrophase in some species. It seems that the general rules of thumb that apply to signal processing do not apply to the analysis of body temperature, possibly because the signal, in the case of body temperature, contains ultradian variation as well as the underlying circadian variation.

Since data loggers have a finite memory capacity, sampling interval is a key determinant of the total logging period, which becomes important in field studies where researchers may wish to measure *T*_c_ over weeks, months, or years. Frequent sampling captures more information but quickly uses up memory. One of the most commonly deployed data loggers is the Thermochron iButton (Maxim Integrated, San Jose, CA, USA). These loggers are mass‐produced to monitor shipments of perishable goods, but they have been widely adopted in the field of animal thermophysiology due to their low cost (approximately USD 100 / unit), small size, long battery life (estimated 10 years according to the manufacturer, depending on the temperature range during use and on sampling rate), and ruggedness (Lovegrove, 2009). However, the most commonly used iButton model (DS1922L) can store only 4092 readings at a resolution of 0.0625°C, which translates to just over 2 weeks of data at a 5‐min interval. The iButtons can be deployed at 0.5°C resolution and take 8192 readings, but that resolution is not acceptable for studies on most homeotherms because the daily range of *T*_c_ is less than 4°C (Hetem et al., 2016), and so only eight points of resolution would be captured. However, logging intervals of 30 or 60 min would permit 12 or 24 weeks of data, respectively, without compromising the accuracy of the estimates of the cosinor variables.

Other commercially available data loggers are specifically designed for animal monitoring, each with their limitations. Like the iButton, the Anipill (BodyCAP, Caen, France; USD 175 excluding peripheral accessories) has a limited memory capacity of 2000 data points at manufacturer‐defined 1‐, 2‐, 5‐, 15‐ or 60‐min intervals. Lifespan is also limited to a maximum of 10 months. The Anipill is equipped with radiofrequency communication and can transmit data to a monitor, which greatly expands its recording capacity, but may not be practical for field use. Another prominent manufacturer is Star‐Oddi, whose products have much larger memory. For example, the DST micro‐T (Star‐Oddi, Gardabaer, Iceland) can record 65,535 measurements, which translates to 227 days of logging at 5‐min intervals, or more than 48 months of recording at 30‐min intervals. However, they cost approximately $600 USD per unit, or six times as much as an iButton, making them prohibitively expensive to adopt in field studies where the risk of logger loss (e.g., due to predation, or death in an inaccessible area) is high. Their utility for long‐term studies is also hampered by their battery life, which is given as 28 months at 10‐min intervals (or 14 months for the smallest available model, the DST nano‐T), but drains even while on standby mode. The larger DST centi‐T has an estimated lifespan of 9 years, but it cannot be used in small animals due to its size. Even so, the long lifespan of the centi‐T loggers is dependent on the logging rate, with actual use averaging six to seven years when sampling at 10‐min intervals, and the longest use being up to 8 years (A. Bjarnason, personal communication, 2022). Given that sampling at 30‐min intervals yields near‐identical estimates of cosinor variables as does more frequent sampling, the large memory capacity of the smaller Star‐Oddi loggers is underutilised and does not justify the additional cost, unless intra‐day (i.e., ultradian) fluctuations in *T*_c_ are of interest.

Because the cosinor approach involves fitting a single component cosinor model to the raw data, the accuracy of the cosinor estimates is impacted by the shape of the *T*_c_ rhythm. The more the *T*_c_ rhythm approximates a cosine wave, the more accurate will be the cosinor estimates of that rhythm. However, not all *T*_c_ rhythms are best described by a sinusoidal waveform. For example, bimodal *T*_c_ rhythms have been described in small rodents like the tree shrew (Refinetti & Menaker, 1992) and mole rat (Finn et al., 2022), and square‐wave rhythms in the fat‐tailed gerbil (Refinetti, 1999) and A/J mouse strain, but not the C57BL/6J strain (Gurkan et al., 2008). In the present study, data from the mouse and barnacle goose did appear more like a square wave than a cosine wave, and the *T*_c_ rhythm in the cheetah was visibly non‐stationary. Although harmonics could be added to the cosinor analysis to achieve a better model fit, in the context of modelling *T*_c_ rhythms, it is not yet clear if multicomponent cosinor models reflect an underlying regulated rhythm, or are simply a mathematical representation of biological noise or masking from activity or environment (Refinetti, 2016). Thus, the single cosinor model presently remains a straightforward and reasonably reliable method of modelling circadian *T*_c_ rhythms.

The presence of sporadic, ultradian events that alter *T*_c_ could contribute to the magnitude of the impact of sampling frequency on cosinor estimates. Ultradian variations naturally exist within *T*_c_ data (Goh et al., 2019), but intra‐day perturbations could also be caused by natural behaviours such as exercise hyperthermia during hunting activity (Hetem et al., 2013) and burrow use in ground‐dwelling animals (Fick et al., 2009; Long et al., 2005). The characterisation of ultradian rhythms is not straightforward, as they often lack true rhythmicity and are more accurately referred to as episodic ultradian events, or basic rest–activity cycles (Blessing & Ootsuka, 2016; Goh et al., 2019). Nevertheless, high‐amplitude ultradian events in *T*_c_ data could lead to inaccurate cosine modelling if the sampling interval used is too large to capture the ultradian event. As an example, mouse *T*_c_ exhibited large and sustained ultradian fluctuations, and the cosinor estimates from the mouse data were the most sensitive of all of the species to longer sampling intervals at the individual level. Similar was true for the rat data. On the other hand, sampling interval had little impact on cosinor estimates in the larger species such as the alpaca, which exhibited infrequent, low‐amplitude ultradian events in *T*_c_. The data in the present study do not allow testing the effect of ultradian characteristics, or of body mass, on cosinor estimates across different sampling intervals, but the proportion of cases that deviated from the original estimates suggest that body mass does not influence cosinor analysis at sampling intervals up to 120 min. Further, the averaged mesor, amplitude, acrophase, and adjusted *R* ^2^ that were derived from mouse *T*_c_ data sampled at 60‐min intervals did not differ significantly from the estimates from the 1‐min data. Sampling intervals can justifiably be increased to up to 60 min in cases where average data are being compiled, because sampling from a population acts as a buffer against the loss in accuracy at the level of an individual.

Based on the results of this study, we advocate that periodogram and cosinor analysis of circadian *T*_c_ profiles is well‐served by a sampling frequency of 30 min, and even 60 min results in only small inaccuracies in the estimates of the rhythm. That interval provides sufficient resolution for reliable estimation of circadian variables while maximising total logging time. Battery life appears to be the biggest constraint with the present logger technology, especially in smaller species. Further improvements in battery and integrated circuit technology may eventually lead to the further miniaturisation of loggers and the extension of battery life and memory.

## AUTHOR CONTRIBUTIONS

**Grace Goh:** Formal analysis (lead); investigation (lead); visualization (lead); writing – original draft (lead). **Kristine Vesterdorf:** Formal analysis (supporting); writing – original draft (supporting). **Andrea Fuller:** Data curation (equal); writing – review and editing (supporting). **Dominique Blache:** Conceptualization (equal); data curation (equal); investigation (supporting); supervision (equal); writing – review and editing (equal). **Shane K. Maloney:** Conceptualization (equal); data curation (equal); investigation (supporting); supervision (equal); writing – review and editing (equal).

## CONFLICT OF INTEREST STATEMENT

The authors declare that they have no conflicts of interest.

## Data Availability

The original data are available at DOI: https://doi.org/10.5061/dryad.1g1jwsv46.

## APPENDIX 2

### 2.1

List of references from which logging intervals were obtained for Figure 1.
